# Stimulus–Response Plots as a Novel Bowel-Sound-Based Method for Evaluating Motor Response to Drinking in Healthy People

**DOI:** 10.3390/s24103054

**Published:** 2024-05-11

**Authors:** Takeyuki Haraguchi, Takahiro Emoto

**Affiliations:** 1 Science and Technology, Graduate School of Sciences and Technology for Innovation, Tokushima University, Tokushima 770-8506, Japan; c612244001@tokushima-u.ac.jp; 2 Division of Science and Technology, Graduate School of Technology, Industrial and Social Sciences, Tokushima University, Tokushima 770-8506, Japan

**Keywords:** bowel sound, BS-based stimulus–response curve, BS-time-domain acoustic features, BS-based stimulus–response plots

## Abstract

Constipation is a common gastrointestinal disorder that impairs quality of life. Evaluating bowel motility via traditional methods, such as MRI and radiography, is expensive and inconvenient. Bowel sound (BS) analysis has been proposed as an alternative, with BS-time-domain acoustic features (BSTDAFs) being effective for evaluating bowel motility via several food and drink consumption tests. However, the effect of BSTDAFs before drink consumption on those after drink consumption is yet to be investigated. This study used BS-based stimulus–response plots (BSSRPs) to investigate this effect on 20 participants who underwent drinking tests. A strong negative correlation was observed between the number of BSs per minute before carbonated water consumption and the ratio of that before and after carbonated water consumption. However, a similar trend was not observed when the participants drank cold water. These findings suggest that when carbonated water is drunk, bowel motility before ingestion affects motor response to ingestion. This study provides a non-invasive BS-based approach for evaluating motor response to food and drink, offering a new research window for investigators in this field.

## 1. Introduction

Constipation is a prevalent disorder that causes difficulties in defecation, abdominal pain, and abdominal bloating, thereby impairing the quality of life. The prevalence of constipation is estimated to be approximately 15% globally [1]. In particular, previous studies have reported that irritable bowel syndrome (IBS) with predominant constipation (IBS-C) is associated with abnormal motility, and it is characterized by an imbalance between segmentation and propulsion [2,3]. Similarly to some Western studies, many colon transit studies in Asian patients with IBS have reported that patients with IBS-C generally have delayed colon transit [4].

Bowel motility can be evaluated mainly with cine-magnetic resonance imaging and colonic transit time [5]. However, these diagnostic tools are complex, non-invasive, and difficult to use routinely. Previous studies have shown that laxatives are prescribed according to the assumption that a direct correlation exists between their use and ease and/or frequency of bowel movements [6]. However, chronic laxative abuse leads to watery stools and anal stenosis [7].

Bowel sounds (BSs) are generated by contractions of the gastrointestinal tract and movement of the mixture of liquid and gaseous contents [8], and they can be detected using a simple, low-cost, non-invasive approach. Bowel motility is evaluated using BS-time-domain acoustic features (BSTDAFs) (e.g., number of BS episodes per minute, sound-to-sound interval (SSI), amplitude, and BS length) [9,10,11,12,13,14,15,16,17,18] extracted from bowel sounds (BSs), which are detected non-invasively. Various BSTDAFs can be used to evaluate bowel motility, and examples include the evaluation of the postoperative intestinal motility recovery state [11,12,13], estimation of the intestinal motility phase during a temporally coordinated cyclic motor pattern known as inter-digestive migrating motor contraction [14], identification of IBS for proof of concept for the use of bowel sound analysis [15], and monitoring the effect of food consumption [16].

Studies have used these BSTDAFs to evaluate the difference in bowel motility before and after eating [15,17] and drinking [9,10] tests, which stimulate/promote bowel motility. In the consumption test for carbonated water [19,20], variations were observed in bowel motility prior to consumption; however, a considerable increase in motility was confirmed after consumption [9,10]. These reports and preliminary experiments show that (i) bowel motility has been statistically evaluated through the increase and decrease in BSTDAFs averaged over different study participants, and (ii) BSTDAFs before and after drinking as well as motor responses to drinking vary among individuals.

However, to the best of our knowledge, no study has investigated the effect of BSTDAFs before drinking on those after drinking. Thus, the aim of this study was to investigate how BSTDAFs before drinking affect those after drinking, as well as to clarify the relationship between bowel motility before drinking and motor response to drinking. Two types of drinks, i.e., water and carbonated water, were used to propose novel BS-based stimulus–response plots (BSSRPs), which incorporate the relationship between BSTDAFs before drinking and the ratio of BSTDAFs before and after drinking. We also used BSSRPs to effectively investigate the effect of BSTDAFs before drinking on motor response to drinking.

## 2. Materials and Methods

### 2.1. Participant Database

This study was approved by the Tokushima University Graduate School of Technology, the Industrial and Social Sciences Science and Technology Course, and the Bioresource Industry Course Research Ethics Committee. All experiments were conducted after the participants were briefed. Informed consent was obtained from all participants, who were confirmed to be free of IBS using the Rome III diagnostic criteria.

We prepared carbonated water as a beverage for consumption, as carbonated water considerably promotes motility more than water does, and we used water as a control. Two databases (DB1 and DB2) obtained from the beverage consumption test were used for the study (see Table 1). To ensure analytical consistency, a 200-mL beverage was administered to participants, as reported in our previous studies [9,10,21].

The drinking test included 5 min of rest before consumption and 10 min of rest after consumption. The participants fasted for approximately 12 h prior to the commencement of the study.

During the drinking test, recordings were performed using an electronic stethoscope (E-scope2, Cardionics Inc., Houston, TX, USA) and stored using a multi-track recorder (R16 Zoom Co., Ltd., Tokyo, Japan or UA-1010 Roland Corp., Shizuoka, Japan). All recordings were performed in complete silence, with the participants in the supine position, and an electronic stethoscope was placed 9 cm to the right of the navel and fixed in a square shape using masking tape. The recording data were acquired at a sampling frequency of 44,100 Hz and a digital resolution of 16 bits/sample. These data were subsequently downsampled to a sampling frequency of 4000 Hz, considering the frequency characteristics of the electronic stethoscope and the frequency characteristics of BSs.

### 2.2. Improved Artificial-Neural-Network-Based BS Detection Method

To detect BSs in the recorded data, we introduced improvements to a method published in 2021 [10] and used power-normalized cepstral coefficients (PNCCs), linear prediction cepstral (LPC) coefficients (LPCCs) [22], and artificial neural network (ANN)-based automatic BS extraction. Using this automatic detection method, the recorded data were first segmented with a segment length of 64 ms and a shift size of 16 ms. The PNCC (20 dimensions) and LPCC (20 dimensions) that were obtained for each segment were input to the trained ANN to obtain its binary output. Herein, the power–bias subtraction process was removed from the PNCC feature extraction. BS segments were detected by applying a threshold to the obtained binary output. Single and consecutive segments were each treated as single BS episodes.

### 2.3. BS-Based Stimulus–Response Plots

The BS episodes detected using the automatic detection method described in the previous section were filtered through a third-order Butterworth bandpass filter with a cutoff frequency of 100–1500 Hz after considering the main frequency band of the BS [23,24]. We used these filtered BS episodes to extract four BSTDAFs that have been commonly used in studies that evaluate intestinal motility from BSs: the number of BS episodes per minute, the sound-to-sound interval (SSI), the BS power, and the BS length.

To investigate whether BSTDAFs before drinking influenced BSTDAFs after drinking, we constructed BSSRPs according to the following procedure:

1. $x_{b}$ was defined as BSTDAFs obtained from BS episodes before intake, and $x_{a}$ was defined as BSTDAFs obtained from BS episodes after intake. The average values of $x_{b}$ and $x_{a}$ were calculated as $\bar{x_{b}}\;and\;\bar{x_{a}}$, respectively.
2. The calculated $\bar{x_{b}}\;and\;\bar{x_{a}}$ were used to calculate the rate of increase, indicating the stimulus response (i.e., motor response to drinking), using Equation (1):
  (1) $$ratio=\bar{x_{a}}/\bar{x_{b}}$$
3. The ratio and $\bar{x_{b}}$ calculated for each participant were used to create a scatter plot with the vertical axis as ratio and the horizontal axis as $\bar{x_{b}}$; the plots are what we refer to as BSSRPs. In addition, to investigate the correlation using the BSSRPs, we used log–log BSSRPs, being double-logarithms of the BSSRPs.

We used this log–log BSSRP to investigate the relationship between intestinal motility before drinking and motor response to drinking.

### 2.4. Statistical Analysis

Correlations were evaluated using Pearson’s product–moment correlation coefficient, which expresses the strength of the linear relationship between two variables. The correlation coefficient may show a high value by chance when the sample is small. Hence, a Student’s *t*-test was conducted with a null hypothesis of no correlation. A correlation was determined to be significant if the *p*-value obtained by the test was *p* < 0.05.

## 3. Results

BSs were automatically detected from the recorded data using the ANN-based improved BS detection method described in Section 2.2. To evaluate the performance of this BS detection method, we used the same database used for the method published in 2021 [10]. The results revealed that the performance of the proposed BS detection system had a sensitivity of 90.58 ± 4.46, a specificity of 91.76 ± 4.61, a PPV of 66.92 ± 8.47, an NPV of 98.45 ± 0.55, a precision of 92.07 ± 3.34, and an F1 score of 76.60 ± 5.89. Note that the database used in the analysis included the data acquired in approximately 50 dB noisy environments.

### 3.1. BSSRP and Log–Log BSSRP Obtained via the Drinking Test

**Figure 1:**
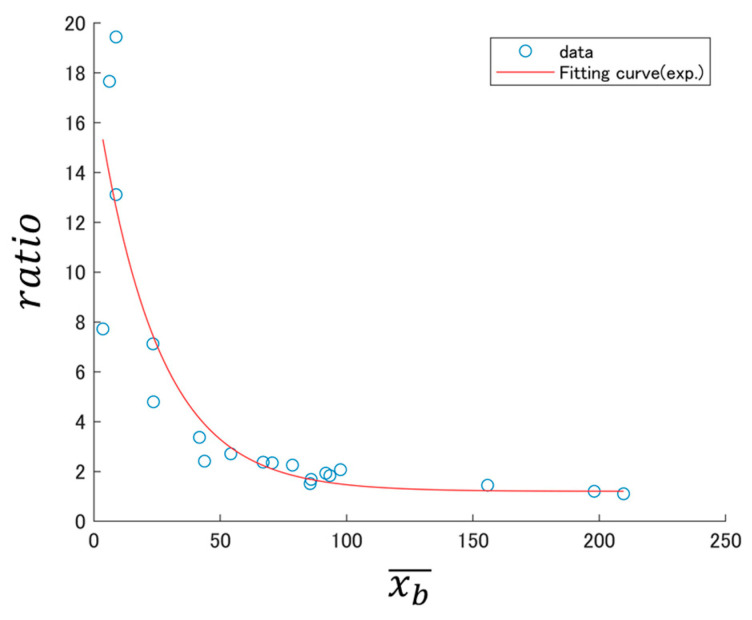
Example of BSSRP obtained from DB1.

As an example, let $\bar{x_{b}}\;and\;\bar{x_{a}}$ denote the average number of BS episodes per minute calculated from the recorded data of 5 min before and 10 min after consuming carbonated water, respectively, for one participant in DB1. Figure 1 shows the BSSRP obtained from the $\bar{x_{b}}\;and\;\bar{x_{a}}$ of all participants in DB1.

As illustrated in Figure 1, the BSSRP decreases exponentially when carbonated water is consumed. Fitting with an exponential function resulted in the equation $ratio=16.4e^{-0.0412\bar{x_{b}}}+1.20$. The adjusted R-squared is used as a measure to evaluate the appropriateness of the model’s approximation. This value can take any number up to one, with values closer to one indicating a more appropriate approximation. For the exponential fitting, the adjusted R-squared was 0.748, indicating that the model provides a good approximation.

**Figure 2:**
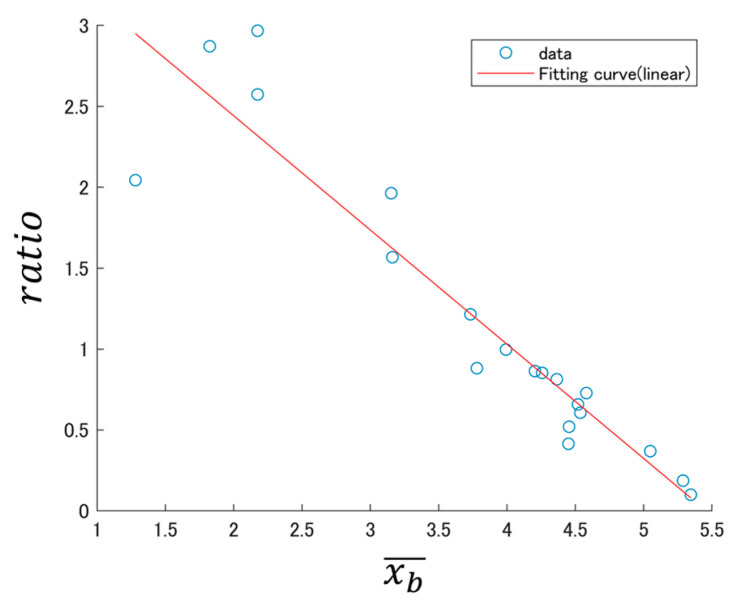
Example log–log BSSRP obtained from DB1.

To further investigate this exponential decline, we present the log–log BSSRP of Figure 1 in Figure 2.

In Figure 2, the log-log BSSRP appears to decrease linearly. Fitting with a linear function yielded the equation $\log\left(ratio\right)=-0.706*log(\bar{x_{b}})+3.85$. The adjusted R-squared was 0.873, indicating that the model provides a good approximation.

The correlation coefficient between log($\bar{x_{b}}$) and log(ratio) is R = −0.938. This trend suggests a strong negative correlation between the two parameters.

### 3.2. Correlation Coefficients of Log–Log BSSRP in Variable Analysis Interval

We examined whether the robust negative associations observed in the log–log BSSRP method when consuming carbonated water could also be observed with other BSTDAF methods, and we tested whether these associations were present in other databases. These examinations were conducted with different analysis intervals. The analysis interval after drinking was the time elapsed from the point of drinking, whereas the analysis interval before drinking was to count time backward from the point immediately before drinking.

Table 2 presents the correlation coefficients and their significance levels obtained using the log–log BSSRP consisting of $\bar{x_{b}}$ and the ratio, with a pre-drinking interval of 5 min and a post-drinking interval of 10 min.

Table 3 presents the correlation coefficients and their significance levels obtained from the log–log BSSRP consisting of $\bar{x_{b}}$ and the ratio, with a 5-min pre-drinking period and a 5-min post-drinking period.

Table 4 presents the correlation coefficients and their significance levels obtained from the log–log BSSRP consisting of $\bar{x_{b}}$ and the ratio, with the pre-drinking interval of 1 min and the post-drinking interval of 1 min. However, we excluded from the calculations the data for one individual for whom BSs were not detected in the 1-min analysis period before and after drinking. Similar measures were taken in subsequent calculations.

As indicated in Table 2, Table 3 and Table 4, no correlation could be confirmed from the R values in the case of cold water consumption (DB-2), which was considered to cause a relatively lower bowel stimulation. In DB-1, BSTDAFs had a negative correlation even when the analysis interval was shortened. The number of BS episodes per minute and the BS length tended to have a strong correlation compared to those of other BSTDAFs for the consumption of a beverage that stimulates the intestines. In particular, the number of BS episodes per minute showed the strongest negative correlation in all BSTDAFs.

Additionally, the S–S interval and the no. of BS episodes/min showed similar trends. Moreover, the correlation coefficient changed as the interval before and after the beverage consumption changed. In particular, the BS power tended to change significantly, which clearly indicates that it is affected by the length of the interval before and after drinking.

### 3.3. Analysis Interval Length and Variance for Log–Log BSSRP for Each Participant

The results in Table 2, Table 3 and Table 4 indicate that the length of the interval (analysis interval) before and after consuming the beverage affected the correlation coefficient. The BSTDAF generally fluctuated with time. Therefore, we investigated the extent to which the plot points for each participant varied owing to the difference in the length of the analysis interval. An examination was conducted using the number of BS episodes per minute, which was strongly correlated in Table 2, Table 3 and Table 4.

**Figure 3:**
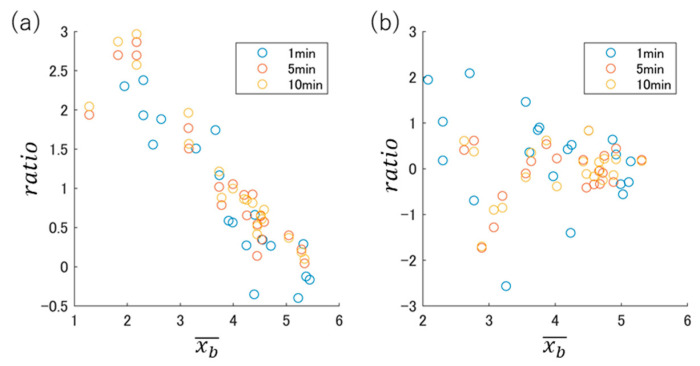
Relationship between log–log BSSRP and analysis interval length in (**a**) DB-1 and (**b**) DB-2: 1 min interval before and after drinking, 5 min interval before and after drinking, and 10 min interval before and after drinking.

The log–log BSSRP results obtained using DB1 and DB2 are plotted in Figure 3. Considering the plot points obtained from the data of 5 min before consumption and 10 min after consumption as a reference, the plot points obtained from the interval before and after consumption (5 min before consumption and 5 min after consumption) were close to each other. The plot points obtained from the intervals before and after ingestion of the beverage (1 min before consumption and 1 min after consumption) tended to be far apart.

To express the variation of plot points caused by the difference in the length of the analysis interval for each participant, the distance on the log–log BSSRP in Figure 3 is defined as follows:

(2) $$r\left(A,B\right)=\sqrt{{\left(log\left(\bar{x_{b,A}}\right)-log\left(\bar{x_{b,B}}\right)\right)}^{2}+{\left(log\left({ratio}_{A}\right)-log\left({ratio}_{B}\right)\right)}^{2}}.$$

**Figure 4:**
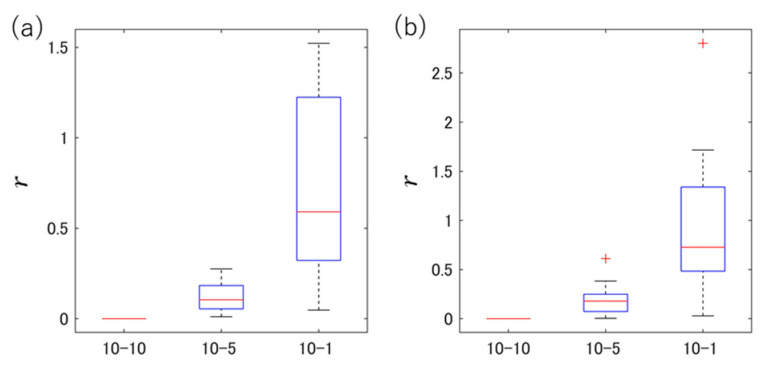
Using each participant database: (**a**) DB-1 and (**b**) DB-2. Relationship between the distance *r* calculated for each participant and each two intervals: 10–10 (analysis interval (5 min before consumption and 10 min after consumption)), 10–5 (analysis interval (5 min before consumption and 10 min after consumption) and analysis interval (5 min before consumption and 5 min after consumption)), and 10–1 (analysis interval (5 min before consumption and 10 min after consumption) and analysis interval (1 min before consumption and 1 min after consumption)).

Herein, the lengths of the two analysis intervals in the participant unit are A and B, respectively. The average values of the BSTDAFs obtained from the pre-consumption intervals are $\bar{x_{b,A}}$,$\bar{x_{b,B}}$, and the increase rate calculated based on the values is ${ratio}_{A}$ and ${ratio}_{B}$. The plots in Figure 4 show the distance *r* obtained from each two intervals: 10–10 (analysis interval (5 min before consumption and 10 min after consumption)), 10–5 (analysis interval (5 min before consumption and 10 min after consumption) and analysis interval (5 min before consumption and 5 min after consumption)), and 10–1 (analysis interval (5 min before consumption and 10 min after consumption) and analysis interval (1 min before consumption and 1 min after consumption)). The distance *r* was obtained for each participant using each participant database, (a) DB-1 and (b) DB-2, and the resulting distribution is represented as a boxplot.

It was confirmed that a larger analysis interval resulted in a smaller dispersion of the BSSRP plot points, regardless of the drink consumption. The participants had 0 BS detections in an analysis interval of 1 min; thus, an analysis of a certain length was thought to be ideal.

### 3.4. Influence of BS Episode Formation and BS Detection Limit on BSSRP

DB1 in Table 2, Table 3 and Table 4 shows that when carbonated water was consumed, the number of BS episodes/min showed a high negative correlation coefficient among the BSTDAFs, even in a short analysis interval.

**Figure 5:**
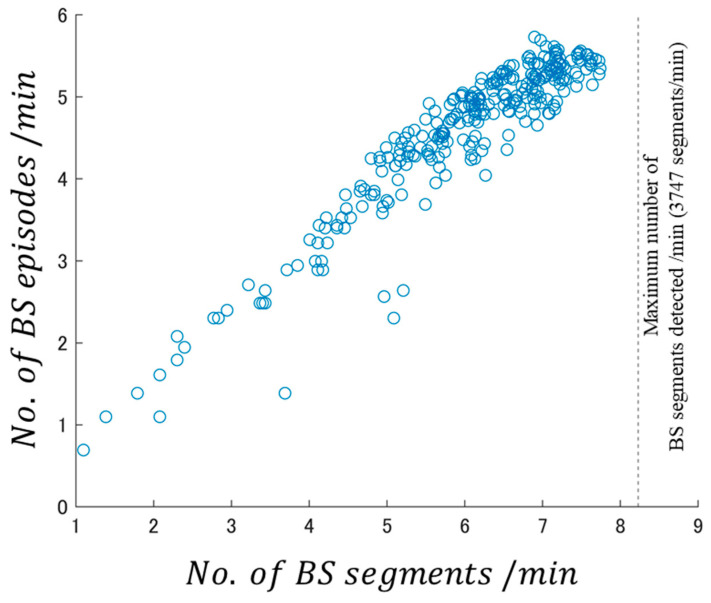
Log–log scatter plot of BS episodes and BS segments obtained by analyzing DB1 in 1 min sub-analysis intervals. Dashed line: maximum number of BS segments detected/min (3747 segments/min).

As defined above, a BS episode consists of a single BS segment or connected BS segments. However, here, we discuss whether this result was caused by (i) a BS segment becoming an episode or (ii) the BS detection limit. Therefore, a sub-analysis interval was created by dividing the analysis intervals into equal intervals of 1 min in length. We investigated the relationship between the BS segments detected in the sub-analysis interval and the number of BS episodes. Figure 5 shows the relationship between the number of segments and the number of episodes obtained by analyzing DB1 in sub-analysis interval units as a scatter plot.

The log–log scatter plot of the number of BS episodes and the number of BS segments per minute (Figure 5) shows that a strong correlation (R = 0.943) existed between both variables. Additionally, the correlation coefficient when logarithm processing was not conducted in the scatter plot of Figure 5 was R = 0.829. Although the number of BS segments per minute did not reach the detection limit, the linear relationship between the number of BS segments per minute and the number of BS episodes slightly weakened only after the number of BS segments per minute was approximately $e^{7.8}≒2440$. In such areas, even though many BS segments were detected, the BS episodes were few, and although bowel sounds were detected, the number of BS episodes was small. Hence, motility was underestimated. Therefore, we conclude that the log–log BSSRP is slightly affected when the number of BS segments is extremely close to the maximum number of BS segments that can be detected by the BS detection system.

**Figure 6:**
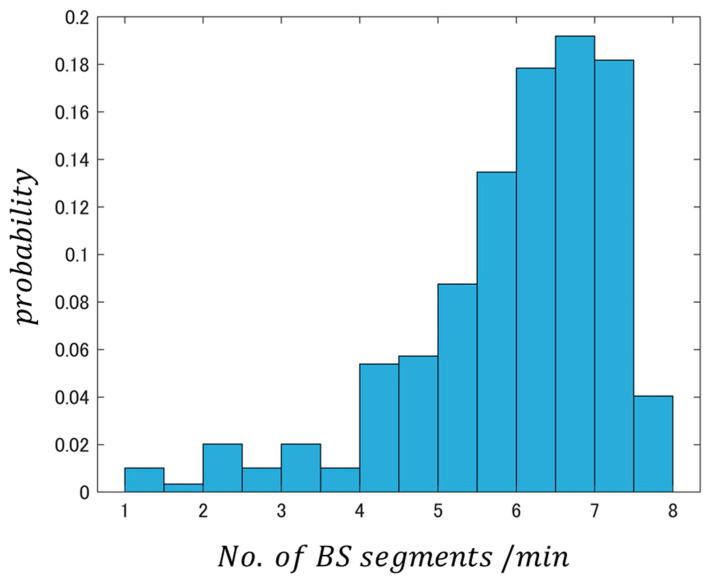
Occurrence frequency histogram showing logarithm of BS segments/min obtained by analyzing DB1 in 1 min sub-analysis interval units.

Next, we investigated the occurrence of cases in which the relationship between the number of segments/min and the number of episodes/min slightly weakens. Figure 6 (based on data from Figure 5) shows an occurrence frequency histogram where the horizontal axis is the number of segments/min.

Figure 6 shows a plot of the number of BS segments/min and the occurrence probability. The figure shows that the occurrence probability of $e^{7.8}≒2440$ or more, where the linearity slightly decreases in Figure 5, is less than approximately 4%. This result suggests that the effect of converting BS segments to episodes on BSSRP is extremely small, even when using a 1-minute analysis interval.

**Figure 7:**
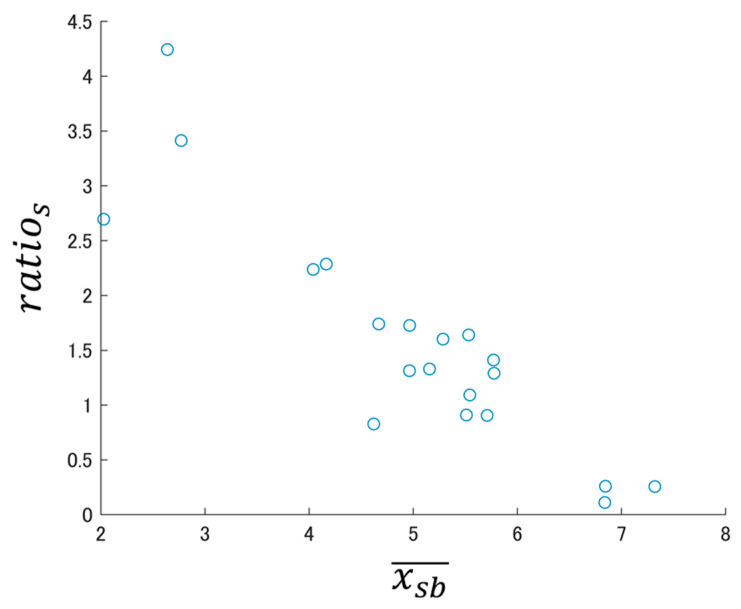
Number of segments/min log–log BSSRP obtained from DB1. Analysis interval (5 min before consumption, 10 min after consumption).

Next, we confirmed whether we could obtain the same tendencies when using the analysis interval (5 min before consumption and 10 min after consumption) to create the BSSRP using the number of BS segments/min as those obtained when using the number of BS episodes/min. Figure 7 shows the log–log BSSRP based on the average value $\bar{x_{sb}}$ of the number of BS segments/min obtained from the pre-consumption interval as the horizontal axis and the increase rate $ratio_{s}$ calculated from $\bar{x_{sb}}$ and the average value $\bar{x_{sa}}$ of the number of segments/min obtained from the post-consumption interval as the vertical axis. Figure 7 shows that the correlation coefficient between $\bar{x_{sb}}$ and $ratio_{s}$ is R = −0.899, confirming that there was a strong negative correlation similar to the log–log BSSRP created with the number of BS episodes/min.

**Figure 8:**
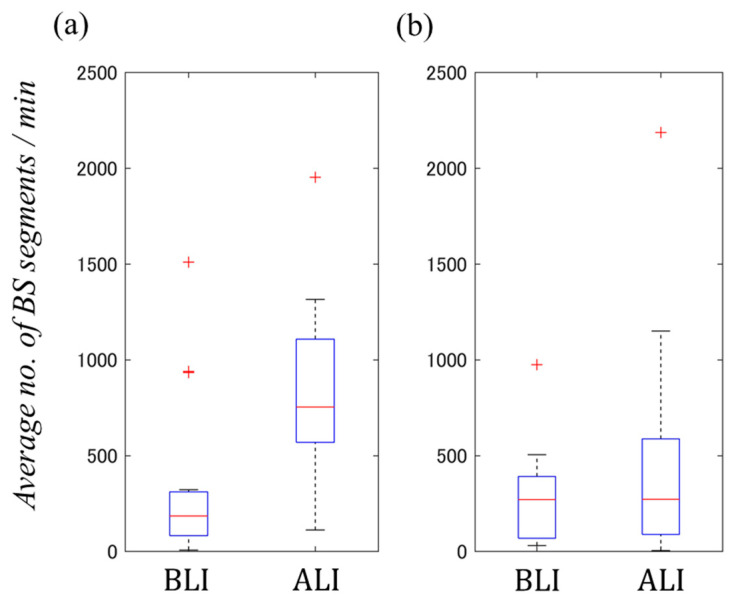
Comparison of differences in average no. of BS segments/min obtained from two databases, (**a**) DB-1 and (**b**) DB-2, before drink consumption (BLI) and after drink consumption (ALI).

Furthermore, we investigated whether the results illustrated in Figure 7 were due to the detection limits of the BS automatic detection system. Similarly, we used the BS segment to conduct an investigation. We calculated the average number of BS segments/min $\bar{x_{sb}},\;\bar{x_{sa}}$ from the analysis interval (5 min before consumption and 10 min after consumption) of the two databases (DB1 and DB2); the results are presented in Figure 8.

Figure 8 shows that the maximum values of $\bar{x_{sb}},\;\bar{x_{sa}}$ obtained from each DB were approximately 2250 segments/min, which was less than 60% of the average number of BS segments/min (3747 segments/min). The latter value is the detection limit of this system after the consumption of carbonated water, which increases motility. Therefore, the high negative correlation observed in the log–log BSSRP was not caused by the detection limit of the BS automatic detection system preventing the detection of BSs that should have been detected.

## 4. Discussion

Using the BSSRP proposed in this work, we investigated the relationship between BSTDAF before drinking and after drinking using a drinking test.

The results of constructing approximate curves for BSSRP and log–log BSSRP show that the adjusted R-squared for BSSRP is 0.748, confirming a good approximation. The adjusted R-squared for log–log BSSRP is higher at 0.873, indicating an even better approximation. Accordingly, it can be concluded that log–log BSSRP provides higher accuracy in predicting stimulus responses, and practical applications are expected to benefit from this method.

We found that when carbonated water was consumed, a high negative correlation existed between both variables in the log–log BSSRP, which indicates that BSTDAFs before consumption influenced BSTDAF after consumption. We also confirmed that this correlation was not affected by the detection limit of the BS automatic detection system. Among the BSTDAFs, the number of BS episodes per minute had the highest correlation coefficient, suggesting that this acoustic feature had the strongest relationship with mobility before consumption. Meanwhile, we found that the correlation coefficient decreased considerably when cold water was consumed. Previous reports have shown that consuming carbonated water stimulates and promotes intestinal motility more strongly than consuming cold water [10,19,20,25,26]. The consumption of such highly stimulating carbonated water would produce a consistent and predictable response in terms of the number of BS episodes per minute. This suggests that the motor response to drinking depends on the strength of the stimulus and the state of motility before consumption.

In this study, we used an analysis interval of up to 10 min after drinking when constructing the log–log BSSRP. Sufficient enhancement of intestinal motility due to carbonated water stimulation was confirmed in this interval [10,21]. A study showed that carbonated water consumption leads to faster and more reproducible intragastric tablet disintegration than tap water consumption [19]. In other words, the motor response to carbonated water is strong and fast, which suggests that this analysis interval was effective.

The log–log BSSRP proposed in this study showed that as the number of BS episodes per minute before carbonated water consumption increased, the increase rate in the number of BS episodes per minute after consumption decreased. In living organisms, heart rate and body temperature exhibit similar trends. Heart rate increases as exercise load increases [27,28]. However, maintaining a high heart rate reduces the left ventricular systolic function and causes tachycardia-induced cardiomyopathy [29]. As is well-known, the maximum heart rate is considered when guiding exercise intensity [30]. Meanwhile, body temperature is related to human energy metabolism and changes with exercise [31], psychological stress [32], and immune response [33]. Higher energy metabolism and higher body temperature have been shown to improve physical performance [34] and immune activation [32]. However, when the core body temperature exceeds 42 °C, cellular proteins are destroyed, which leads to death [35]. These results indicate how temperature and heart rate are maintained at upper limits by homeostasis. Similarly, intestinal motility has an upper limit due to homeostasis, and if intestinal motility is enhanced before stimulation, then intestinal motility after stimulation is suppressed.

To date, BS analysis before and after meal consumption [15,17,18,36] and BS analysis before and after drinking [9,10,21] have been conducted. Studies have reported that intestinal motility is statistically significantly enhanced after eating and drinking. These reports are based on the characteristics of BSs before and after consumption, which are thought to stimulate intestinal motility. In the future, it is expected that the use of a log–log BSSRP in food and drink consumption will yield a more appropriate evaluation of an individual’s motor response to food and drink.

Research by Craine [17] and Marshall [15] suggests that it is possible to distinguish between IBS and non-IBS by evaluating the response to food based on the acoustic characteristics of BSs. Future research on IBS identification based on BSs will likely provide a more appropriate evaluation of an individual’s motor response to food and drink.

This study had some limitations. An analysis interval of up to 10 min after drinking carbonated water was used when constructing the log–log BSSRP. It will be necessary to examine whether this analysis interval is also valid for other drinks.

A drink consumption test was conducted in this study; however, it will be necessary to investigate whether similar effects are observed when “foods that stimulate the intestines” other than carbonated water are consumed. Furthermore, it is important to investigate the relationship between intestinal motility and factors potentially related to gastrointestinal motility, such as carbonation level, beverage volume, and their combined effect. Further investigation will be required to determine the mechanism by which intestinal motility before drinking influences motor response to drinking. We will address these research questions in future studies.

## 5. Conclusions

We conducted carbonated water consumption and water consumption tests using BSSRPs constructed according to BSTDAFs. The results showed that when carbonated water is consumed, intestinal motility before consumption affects motor response to carbonated water. Meanwhile, no such effect was observed when cold water was consumed. These results suggest that the motor response to drinking depends on the strength of the stimulus and the state of intestinal motility before consumption. Therefore, the proposed BSSRP is expected to open up new paths for evaluating the motor response to food and drinks. 

## Figures and Tables

**Table 1:** Participant database.

	Beverage	Temperature	Rome III Diagnostic Criteria	Participant Data				
				Number of Persons	Age	Height [cm]	Weight [kg]	BMI
DB1	Carbonated water	10 °C or less	Healthy	13 men and 7 women	32.35 ± 8.51	168.1 ± 7.97	59.70 ± 8.43	21.06 ± 2.02
DB2	Water							

**Table 2:** Correlation coefficient calculated using log–log BSSRP (5 min before beverage consumption and 10 min after beverage consumption).

	DB-1		DB-2	
	R	*p*	R	*p*
BS power	−0.597	0.005	−0.312	0.180
BS length	−0.755	0.000	0.232	0.325
No. of BS episodes/min	−0.938	0.000	0.299	0.200
S–S interval	−0.886	0.000	0.309	0.186

R: correlation coefficient; *p*: *p* value.

**Table 3:** Correlation coefficient calculated from log–log BSSRP (5 min before drinking and 5 min after drinking).

	DB-1		DB-2	
	R	*p*	R	*p*
BS power	−0.490	0.028	−0.179	0.450
BS length	−0.626	0.003	0.318	0.172
No. of BS episodes/min	−0.924	0.000	0.278	0.235
S–S interval	−0.855	0.000	0.288	0.219

R: correlation coefficient; *p*: *p* value.

**Table 4:** Correlation coefficient calculated based on log–log BSSRP (1 min before drinking and 1 min after drinking).

	DB-1		DB-2	
	R	*p*	R	*p*
BS power	−0.518	0.023	−0.541	0.014
BS length	−0.658	0.002	−0.535	0.015
No. of BS episodes/min	−0.925	0.000	−0.311	0.181
S–S interval	−0.858	0.000	−0.409	0.073

R: correlation coefficient; *p*: *p* value.

## Data Availability

Data are unavailable due to privacy or ethical restrictions.
